# The Impact of a Physiotherapy-Led Virtual Clinic in a South Australian Hospital: A Quantitative and Qualitative Investigation

**DOI:** 10.3390/healthcare13172185

**Published:** 2025-09-01

**Authors:** Mark Jarrett, Matthew Beard, Saravana Kumar

**Affiliations:** 1 Central Adelaide Local Health Network, Adelaide, SA 5000, Australia; mark.jarrett@sa.gov.au (M.J.); matthew.beard@sa.gov.au (M.B.); 2 IIMPACT in Health, Allied Health & Human Performance, University of South Australia, Adelaide, SA 5001, Australia

**Keywords:** advanced practice physiotherapists, physiotherapy-led, specialist outpatient services, spinal clinic, spinal service

## Abstract

**Background**: As means of addressing ongoing challenges in accessing publicly funded specialist care, new models of care have been trialled. One such approach is using physiotherapists in advance practice roles, who in collaboration with other health professionals, act as an initial orthopedic point of contact and coordinate care. This research investigated the impact of a model of care, the Spinal Virtual Clinic Model, implemented for the first time in South Australia, using advanced practice physiotherapists in a large metropolitan hospital in South Australia. Although formally named the “Spinal Virtual Clinic” by the health service, this model does not involve direct patient contact and differs from traditional virtual or telehealth clinics. Instead, it is best understood as a physiotherapy-led referral triage and management service. **Methods**: This research was conducted in two stages. Stage 1 was a retrospective clinical audit of sequential patients triaged to the Spinal Virtual Clinic, as well as a follow up audit to capture any subsequent engagement with the Orthopaedic Spinal Service following the initial Spinal Virtual Clinic correspondence. Data were descriptively analysed. In Stage 2, semi-structured interviews were conducted with patients from the Spinal Virtual Clinic to explore their perspectives on this model of care. The interviews were transcribed verbatim and independently analysed using thematic analysis. The sequential use of quantitative and qualitative approaches enabled us to both describe engagement with this model of care and better understand the underlying perspectives. **Results**: Three hundred and nine referrals were triaged to the physiotherapy-led spinal virtual clinic over a six-month period from 1 January 2021 to 30 June 2021. Majority of referrals were triaged as low acuity did not need formal spinal specialist review and could be managed safely in primary care. Therapist-led active management strategies (80.8%), trial of neuropathic medication (35.6%) closely followed by advice regarding targeted spinal injections (foraminal and epidural), were the most common conservative management strategies recommended. Only a small proportion needed surgical review. Interviews with eleven patients revealed that while many valued the convenience, timely advice, and reassurance offered by the service, others expressed confusion about the referral process and disappointment at not seeing a specialist. A key recommendation identified was improved communication, including providing patients with direct feedback alongside general practitioner correspondence. **Conclusions**: This research, underpinned by quantitative and qualitative research, has showcased the potential of this model of care, the spinal virtual clinic, to have a positive impact on improving access and reducing the burden on the health system for low acuity patients. As historical models of care become unsustainable and obsolete, alternative models of care can be implemented in health care settings where outpatient demand significantly exceeds capacity.

## 1. Introduction

Challenges in affording timely and efficient access to healthcare have been well documented both in Australia [1,2] and internationally [3,4], particularly in countries that seek to provide equitable access to healthcare [5]. This is particularly exacerbated with access to specialist outpatient services [5,6,7] with coexisting delays in subsequent wait times for surgery which have been further compounded by the COVID-19 pandemic [8,9].

The demand for access to publicly funded specialist care is high, with often prolonged wait times to an initial orthopedic consultation. Models of care that utilise physiotherapists, as well as other allied health professionals in advanced practice roles have been utilised in a variety of clinical settings including emergency departments [10], specialist outpatient clinics [11,12] and primary care [13]. In Australia, advanced practice physiotherapists (APP) work in roles that are within the scope of physiotherapy but through custom and practice have been performed by medical specialists. These advanced practice roles may require additional training, as well as significant professional experience and competency development [14]. APP have demonstrated high levels of diagnostic accuracy [15], have shown to be cost effective across multiple clinical settings when compared to usual medical care [16], and provide increased accessibility and reduced wait times for access to specialist consultation [17]. Virtual models of care like telehealth have also been embraced for their ability to improve equity of access by removing geographical and physical barriers to accessing care [18], particularly in the context of COVID-19 [19]. Telehealth has demonstrated high levels of consistency between face-to-face and telehealth assessment [20], as well as high levels of patient and caregiver satisfaction [21].

Despite significant research and investment in this area many patients across multiple specialist outpatient services still experience significant delays in accessing care both in Australia [5] and internationally [4], particularly for non-urgent cases [22]. In South Australia, similar challenges are evident with historical wait times for non-urgent outpatient specialist appointments, in some cases exceeding 16 years for plastic and reconstructive surgery [23]. There are numerous reasons for these gaps in health service delivery including: the number of specialists available and the hours they work in the public sector, better remuneration packages in the private sector, and lack of adequate resources in the public sector.

**Figure 1:**
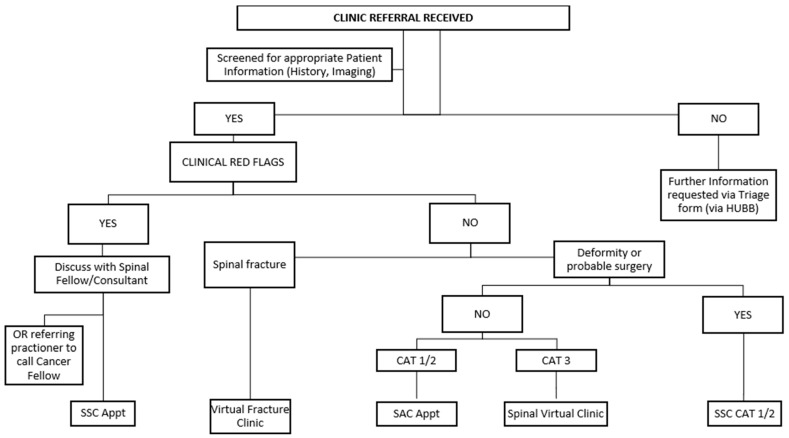
Orthopaedic Spinal Service model of care. CAT: triage category, SAC: spinal assessment clinic, SSC: spinal surgical clinic.

The Orthopaedic Spinal Service at the Royal Adelaide Hospital in Adelaide, Australia, provides a specialised secondary care consultative service, with most referrals involving outpatients with low back pain who have not made satisfactory progress in primary care. The model of care is outlined in Figure 1. These clinics are led by spinal surgical consultants, spinal fellows, and APP. More than 95% of referrals to the clinics are received from general practitioners, servicing a catchment area covering the Adelaide metropolitan area and country South Australia (approximately 980,000 km^2^) with total population of approximately 1.75 million people [24].

In the South Australian spinal outpatient triage system, the scoring system for each category relates directly to acuity and potential for clinical deterioration. Category 1 refers to urgent referrals requiring specialist assessment within 30 days (e.g., progressive myelopathy, acute radiculopathy with motor deficit, spinal tumour, acute fracture, infection etc.). Category 2 includes semi-urgent cases where care should be provided within 90 days (e.g., radiculopathy with stable motor signs). Category 3 is for routine referrals expected to be seen within 365 days (e.g., chronic back pain or radicular leg pain without neurological deficits) [25].

APP have been embedded with the Orthopaedic Spinal Service since 2007 operating as the Spinal Assessment Clinic (SAC). Seven APPs (of varying part-time FTEs) contribute to the delivery of the SVC (as part of their broader case load). The APP coordinate clinical triage of all spinal referrals [26], as well as provide initial orthopedic contact assessments for non-traumatic referrals from primary care. In early iterations, the APP provided an initial contact for low acuity referrals (Category 3) from the service’s long-term waitlist. In more recent years the Spinal Assessment Clinic has evolved to provide an initial point of contact for primarily Category 2 and some Category 1 referrals due to increased work demand, thereby providing timely access to care and supports the work of the spinal surgeons.

The Spinal Virtual Clinic Model: As an alternative to referrals triaged as Category 3 being allocated to a lengthy waitlist for a formal appointment, the APP embedded within the Orthopaedic Spinal Service developed and implemented an alternative clinical pathway—referrals were instead assigned to a physiotherapy-led Spinal Virtual Clinic (SVC). In this role, the APP undertakes a desktop review of the referral, along with the patient’s electronic medical record, which includes relevant outpatient and inpatient clinical notes, pathology results, and radiology reports available within the public hospital system. The APP then reviews the patient’s imaging from both internal and external radiology providers. Finally, the APP provides customised clinical advice in a summary to the referring doctor via the electronic medical record system to guide management in primary care. The APP’s role is to facilitate clinical pathways and ensure patients are appropriately classified and referred to the correct service, without any direct contact (in person or digital) with the patients. It is important to note that while this model is formally referred to as a “Spinal Virtual Clinic” by the implementing health service, it differs from conventional definitions of virtual care (e.g., those involving telehealth or direct patient interaction). Rather, it functions as a physiotherapy-led referral triage and management service.

For this cohort of patients with low likelihood of requiring surgical intervention, the goal of this process has been to facilitate early engagement with general practice in absence of a formal face-to-face appointment with the provision of timely decisions and advice regarding conservative management options in primary care. It also opens a communication pathway with the patient’s referring doctor for further input and advice from the Spinal Service via a dedicated service email. The provision of targeted health information with primary care support has been considered in other diagnostic groups. A systematic review investigated digital health technology to support healthcare provider communication in fragility fracture recovery [27]. The included five randomised controlled trials which found digital health supported interventions were twice as effective compared with standard care.

Preliminary observational evidence within our service suggested that this model of care, offering an alternative pathway for low acuity referrals, helped reduce long term appointment wait times and improved the flow of referrals to the surgeon led Spinal Surgical Clinic (SSC). To build on these initial observations, the study was designed to address a key knowledge gap through two sequential stages. Stage 1 examined: (1) what are the clinical activities, management strategies and safety outcomes associated with the SVC? Clinical activities examined included referral, imaging, medical history, and customised advice provided to the referrer. Management strategies included the type and breadth of strategies and/or advice contained in the customised correspondence sent to the referrer. Safety outcomes included re-referral rates (i.e., subsequent referrer contact) and any activity involving the spinal service in the six months post-referral (e.g., re-referral, subsequent outpatient appointments, ED presentations, or an index procedure). Stage 2 addressed: (2) what are patients’ perspectives and experiences of receiving care through the SVC model?

## 2. Materials and Methods

### 2.1. Research Design

This research utilised sequential quantitative and qualitative research paradigms to acquire data in two separate stages. Stage 1 involved a clinical audit of patient referrals triaged to the SVC. Following this, as part of Stage 2, a qualitative descriptive approach involving semi-structured interviews was undertaken with a small subgroup of patients sampled from the original cohort of patients from Stage 1 to explore patient perspectives on management of their referral via the SVC.

### 2.2. Stage 1: Audit

Study design: The first stage of the research used a retrospective clinical audit of sequential patients referred to the Orthopaedic Spinal Service of the Royal Adelaide Hospital. The referrals were primarily from general practice, and a small number of internal hospital referrals from other specialties who were referred to the service during a six-month period (1 January 2021 to 30 June 2021) and subsequently triaged to the SVC.

Inclusion/exclusion criteria: All patients ≥18 years of age who were referred to the Orthopaedic Spinal Service either by their general practitioner or via an internal hospital referral were included. These referrals were triaged as non-urgent, benign disorders where serious spinal pathology was not suspected, and surgical interventions considered unlikely; previously triaged to a Category 3 waitlist. Completion of a mandatory template for referrals is required for referral acceptance by the service [26]. This scoring system for referrals is imbedded within a mandatory template that also captures additional relevant information, namely response to injection therapies (if undertaken), current pharmacotherapy, spinal imaging and medical history. All clinical metrics then guide the formal triage process, with decisions that are documented. This process is the responsibility of the Lead Physiotherapist, with spinal consultant oversight. Those referrals that do not meet the defined service criteria or require other specialty input are returned to the referring medical officer. At the time of this research was undertaken, a systemized paper-based triage was in place, but this has now been replaced by an electronic triage system (SRMS), but decision-making elements remain unchanged. Excluded referrals for the SVC included those that required more urgent care (Category 1 or 2) at triage, those that required surgical consultation or review in a Spinal Fracture Clinic, and those not accepted due to incorrect referral details or insufficient referral information.

Data collection: The SVC was undertaken by one of the APP working in the Orthopaedic Spinal Service. A customised audit tool was developed to record the clinical activity, management actions, and advice provided as an outcome of the SVC. In addition to demographic data, re-referral and discharge rates, wait times, complaints, and any subsequent email communication between the referrer and Orthopaedic Spinal Service in response to the SVC management were recorded. The overall time spent by the APP actioning each SVC referral was also recorded. The dataset was collated in a study database using a bespoke Microsoft Excel spreadsheet, and unique study identification numbers ensured patient data were de-identified. Microsoft Excel was used to descriptively summarise the collected data.

As an additional component of the audit, for all patients included in the study, a retrospective audit of their state-based public hospital electronic medical records, that captured secondary and tertiary services was undertaken (up to and including 31 December 2021) to capture any subsequent engagement with the Emergency department and/or Orthopaedic Spinal Service following the initial SVC correspondence. This included additional communication/ re-referral to the service, further SVC correspondence sent to the referrer, and any subsequent appointments in Orthopaedic Spinal Service, emergency department presentations, or subsequent spinal surgeries or procedures. The goal of this additional review was to capture any acute admissions that may have been incorrectly triaged or who may have had an adverse outcome. The research team did not have access to primary care or private specialist records. The above dataset was collated into a separate study database Microsoft Excel spreadsheet using the unique study identification numbers.

### 2.3. Stage 2: Semi-Structured Interviews

Study design: Qualitative Descriptive (QD) methodology [28,29] was employed to guide the second stage of the project, which aimed to explore patient experiences and perspectives of the SVC. QD is particularly well suited for applied health research where the goal is to obtain a straightforward, comprehensive summary of events or experiences in participants’ own words. Unlike other qualitative approaches that are more interpretive or theory-driven, QD allows for a low-inference account of the phenomenon, making it ideal for capturing nuanced yet practical insights into service delivery. Given the pragmatic nature of the SVC and the need to inform real-world clinical practice, QD provided an appropriate methodological framework to describe what occurs in this setting and how it is perceived by those using it.

Recruitment: Non-probability purposive sampling targeted patients from the SVC cohort identified in Stage 1. As part of purposive sampling, quota sampling of patients from different ages, geographical locations (metropolitan and regional), and diagnostic groups was undertaken. Patients were invited to participate in this research. A well-recognised form of sampling in qualitative research, purposive sampling is ideal in selecting those patients who are available and have a ‘story to tell’ [28,29]. An estimated sample size of 6–12 participants was deemed adequate to capture diverse perspectives on the topic within a relatively homogeneous participant group, while remaining feasible within the study timeframe.

Procedures: Patients were contacted via phone/email by an administrative staff member and not a clinician involved in the patient’s care to avoid issues of coercion and power imbalance. The patients were provided with the background, purpose, ethical considerations (such as consent, withdrawal, etc.) and all details regarding this research. If the patient consented, a mutually convenient time was organised to conduct a semi-structured phone interview with the principal investigator. A semi-structured interview guide was developed a priori and used during the conduct of the interview. The interview guide was developed based on insights from the literature and aligned with the study objectives. Interview questions broadly covered participants’ awareness of and perspectives on the SVC (“Were you aware you had been referred by your doctor to the Spinal Unit?”, “Did you have positive/negative feelings about the Virtual Clinic process?”), their ongoing care with the GP following the letter from the SVC (“What was the outcome once your doctor received our letter?”), and the progression of their spinal complaint (“How do you feel you’re going now with your spinal complaint, compared to prior to your referral to the Spinal Unit”). All interviews were conducted by the lead researcher (MJ) via telephone between 20 October and 1 December 2021, as the data collection period coincided with COVID-19 restrictions. The interviews were audio-recorded, transcribed verbatim, and thematic analysis used to capture patient perspectives.

Data Analysis: The interviews were analysed using thematic analysis, a well-established analytical process for recognising patterns and identifying themes [30]. Using the process outlined by Green et al. [31], the interviews were examined, and then codes were applied to cluster information into the key themes across the interview cohort. The analysis was led by an independent researcher with expertise in qualitative methods to ensure rigour and trustworthiness, in particular credibility and confirmability of findings. A co-investigator (SK) also collaborated with the independent researcher through regular debriefing and reflective discussions to enhance reflexivity and ensure both were aware of how their perspectives might influence the research process and findings.

Ethics: Ethical approval was sought and granted by the Central Adelaide Local Health Network (CALHN) Human Research and Ethics Committee prior to the commencement of the study (reference number: 14589). Given the nature of the clinical audit and the absence of formal engagement with the patient as part of the SVC process a consent waiver was sought and obtained for the electronic medical record review up to 31 December 2021. Written consent was obtained from all patients participating in interviews in Stage 2 of the research.

## 3. Results

### 3.1. Stage 1: Audit

Three hundred and nine referrals were triaged to the physiotherapy-led SVC over a six-month period from 1 January 2021 to 30 June 2021, generating 317 virtual clinic letters (309 initial and 8 additional/second letters).

Referral characteristics: Of the 309 referrals, 53% were female and 47% were male with an average age of 54.9 years (range 18–90). Across all the SVC correspondence provided to the referring doctor, the vast majority were related to low back pain with or without leg symptoms (63.4%). Neck with or without arm symptoms accounted for a further 16.1% of the cohort. Complete details of referral characteristics is provided in Table 1. The 8.5% categorised as “other”, included incidental haemangiomas on imaging referred for review, coccygeal pain and for isolated limb symptoms without concordant spinal imaging findings.

**Figure 2:**
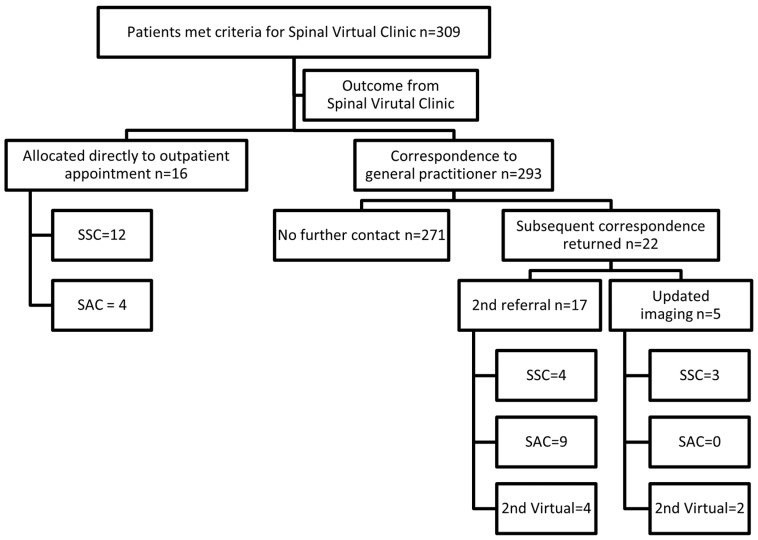
Outcomes following Spinal Virtual Clinic. SAC: spinal assessment clinic; SSC: spinal surgical clinic.

Outcome following SVC: The review led by the APP resulted in 16 patients (5% of the cohort) being identified as requiring a formal outpatient appointment for clinical assessment. The need for an appointment was determined from either further clinical details following a review of the referral and electronic medical records, or key radiology findings identified on review of a patient’s imaging. These 16 patients were allocated a formal outpatient appointment: 11 of these in the surgeon-led Spinal Surgical Clinic, and five in the physiotherapy-led Spinal Assessment Clinic. Three of the 16 cases proceeded to an index spinal procedure, which included: two lumbar fusions (one anterior, one posterior) and a multilevel lumbar decompression. Of the remaining 293 cases, further correspondence was received for 22 cases (7.5% of the remaining cohort) within the review timeframe (up to 31 December 2021). Five cases highlighted updated imaging either on requested as part of the SVC process or initiated by the general practitioner independent of the SVC advice. A second referral was received for 17 patients in the cohort with either new or updated clinical information provided. Of the 22 cases for which further correspondence was received, 17 (77%) were allocated a formal outpatient appointment in either the surgeon or physiotherapy-led clinics. The remainder (n = 6) after being reviewed again in the SVC were discharged back to the referrer with a management plan as outlined in Figure 2. There were no re-referrals for missed pathologies such as fractures or tumours after initial management in the SVC.

Summary of advice provided: The advice provided to the referring doctor regarding conservative management strategies often included multiple strategies for management in primary care (see Table 2). Therapist-led active management strategies (80.8%), trial of neuropathic medication (35.6%) closely followed by advice regarding targeted spinal injections (foraminal and epidural), were the most common conservative management strategies recommended. Advice for further investigation included requests for further spinal imaging: GP initiated cervical MRI (available in Australia under Medicare for suspected cervical radiculopathy), standing plain films, flexion/extension plain films; and requests for blood tests including inflammatory markers and myeloma screen. Requests for the referring doctor to initiate a referral to an alternative medical specialty were most commonly to rheumatology (for suspected inflammatory spinal pain), but also included endocrinology (osteoporosis), plastic surgery (suspected carpal tunnel syndrome), orthopaedics (hip osteoarthritis), pain clinic, vascular (suspected vascular origin of claudicant leg symptoms) and dietician (weight loss). The 12.3% of correspondence that included advice categorised as “Other”, included requests for further clarification of patient symptoms (n = 23), recommendations for falls and balance classes, requests for myeloma screen, and advice that the referral be transferred to a brachial plexus clinic.

Criteria for re-referral: For a portion of referrals, inclusion of the re-referral criteria was deemed not required (n = 52) in the correspondence back to the referrer. This included patients who were booked a clinic appointment as a result of the SVC review (n = 16), and those that were recommended referral to an alternative specialty (n = 15). For the remaining correspondence (n = 264), re-referral criteria were included in 80% (n = 212) of the time, but was not included 20% (n = 53) of the time. Patients with axial spine pain without any reported limb symptoms made up 60% (n = 32) where re-referral criteria were not included.

Timeframes: For referrals triaged to the SVC, the average timeframe for the referrals to be processed, reviewed and a correspondence letter completed to the referring doctor was 34.5 calendar days (range 2–89 days). The SVC process, along with creation of an appropriately detailed letter to the referring doctor took an average of 32.4 min (range 20–60 min). On occasions, a spinal consultant review of the relevant radiology was requested. This occurred on 30 occasions (9.5%), and this detail was noted in the correspondence to the referring doctor. This primarily involved patients with atypical imaging findings such as benign tumours where clarification of imaging findings was required and decisions regarding appropriate management plans were needed.

**Figure 3:**
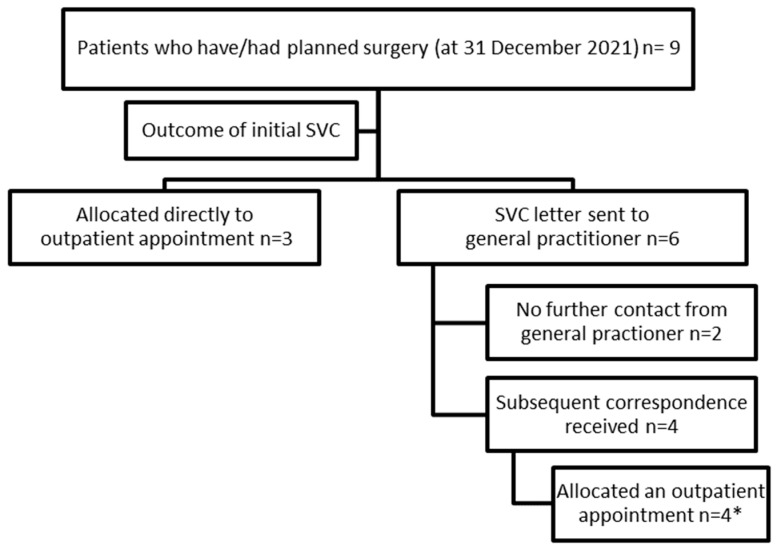
Breakdown of surgical cohort. SVC: spinal virtual clinic. (* *one patient was admitted via ED prior to their planned outpatient appointment)*.

Surgical outcomes of the cohort: A review of all 309 patients’ electronic medical records was undertaken in early 2022 to review clinical activity up to 31 December 2021. Of the 309 patients included in Stage 1, nine patients (2.9%) had either had or been placed on a surgical waitlist for an index spinal procedure (as at 31 December 2021). As outlined in Figure 3, of these nine patients, three were identified in the initial SVC screen as needing formal outpatient review. For one of these patients, a new referral was received by the service with worsening symptoms and was booked in for a review in the Spinal Surgery Clinic (SSC). The second patient was booked a telehealth appointment and then reviewed in the Spinal Surgical Clinic with a plan for conservative management. This patient then had a subsequent presentation via the emergency department with acute cauda equina symptoms and had decompressive surgery. The third patient was referred with worsening low back pain and leg symptoms in the setting of previous two-level disc arthroplasty.

Of the remaining six patients, four were booked formal outpatient appointments in the Spinal Surgical Clinic following further correspondence from their referring doctor as outlined in Figure 3. One of these four patients attended the ED prior to their outpatient appointment with acute-on-chronic low back pain and worsening leg symptoms. The patient was discharged from the ED but re-presented three days later after a fall, with increased leg symptoms and urinary retention. They were admitted and underwent decompressive surgery.

The final two surgical cases, for whom no further contact was received from their GP, were not captured through the SVC. Of these, one was admitted via the ED with acute-on-chronic low back pain and possible acute cauda equina symptoms, five months after the initial referral, and subsequently underwent surgical decompression. The other patient underwent spinal surgery in a local private hospital but was later admitted to the Royal Adelaide Hospital with a cerebrovascular accident as a post-operative complication.

### 3.2. Stage 2: Semi-Structured Interviews

Eleven patients consented to be included in Stage 2 of the research project and to be interviewed regarding their insights and perspectives of the management of their referral via the SVC. The patients were of varying age, gender, employment status and with a variety of presenting complaints (Table 3).

All interviewed patients were aware that they had been referred to the Spinal Service, whether that be by their general practitioner (GP) or via a referral initiated internally following an emergency department presentation. Despite the mix of clinical presentations, there was a clear delineation between patients that could see significant benefit in the SVC model (prompt access, reassurance, non-surgical options for management in primary care, etc.) and those that felt they were being denied access to an appointment, and perceptions of surgery being the outcome that was required. Overall, there was generalised agreement on the value of clear communication to the patient regarding the SVC model. The following section summarises these findings.

Understanding the referral: This theme relates to patients’ understanding of their referral to the service. All patients were aware that they had been referred to the Spinal Service. Most of the cohort were referred to the service by their GP (n = 9), with the remaining (n = 2) having their referral initiated following presentation to the Royal Adelaide Hospital Emergency Department. There was varied understanding by patients of how their referrals had been managed, with only a few patients aware that they had been triaged to the SVC. The majority were unclear about being allocated to the SVC instead of a waitlist for a face-to-face appointment, with interviewed patients describing their understanding as “not-urgent enough [P6]” or “didn’t qualify (for an appointment) [P8]” to “been declined [P5]” and “I never heard anything [P7]”.

GP engagement with management plan: This theme relates to the follow-up actions of the GP following the provision of customised conservative management advice. Several patients reflected that they had received no information regarding the advice provided, but only that “didn’t have a place for me [P4]”. Patients reflected that there was often a lack of communication between the GP and patient. Sometimes this was a result of the patient having changed GP [P8], or the correspondence not being discussed with the patient: “I have seen her (GP) like twice after that, but I never had a chat about it [P3]”.

Experiencing the SVC: This theme relates to the patients’ insights into the SVC. Collectively this cohort indicated mixed perspectives, with some positive, although this was not universally shared. Patients reflected on the convenience and timely management offered via the SVC: “it’s fantastic. It saved me all the problems of getting to town [P5]” and “it’d be better to send it back to the doctor for advice on what to do. Waiting 3–4 years, I mean some people are in severe pain [P6]”. Others felt that the SVC offered them prompt reassurance: “it’s not ready for an operation, so that’s awesome, so let’s try other stuff [P10]”, and also a pathway to better understand options for their condition: “you have a problem obviously but we can manage it this way or that way [P2]”, and “you know, there’s other ways that I can deal with this [P10]”. Conversely, several patients were disappointed with having their referral declined and this was evident in both the patients referred by the GP: “I was a bit disappointed when I got the letter saying I’d been declined [P5]” or via the emergency department: “having been to the A&E, and I literally could hardly move, then to be told that I was considered not worthy to be seen by the back unit, I was very disappointed [P11]”. One patient reflected that “if I had a letter come to me to say that there is no need for surgery … I’d be fine with that [P7]”.

Living with the condition: This theme relates to patient perspectives on the management of their condition and their insights into the status of their symptoms. There was a broad cross section of perspectives on conservative management, with some patients keen to explore these options in preference to surgery: “so I wouldn’t mind going down that avenue, and I’d go down that avenue before I’d have an operation anyway [P10]”, whereas other patients were adamant that conservative management was unhelpful: “to be sent there for physio with the way I am now, I just think, I would just be in more pain, if you know what I mean [P4]”. Overall, most of the patients reflected on some perceived improvement in their symptoms: “I just keep doing my exercises, and I feel it’s so much better [P6]”, “my leg symptoms seem to have cleared up since I had the cortisone [P11]”, but some patients did describe their symptoms as unchanged: “no, it’s the same, I still have to take two pain killers for every day [P3]” or a bit worse: “no, it’s getting a little bit worse. And I don’t know if that’s the stress I’m under [P10]”.

Reimagining the SVC. This theme centres around the patient insights into the SVC as a pathway for their referral, possible future referrals and their reflections on how the SVC could be improved. Several of the patients were positive about the SVC process reflected on the benefits of timely access to review information: “at least you’re letting me know what’s going on [P2]” and “It made me feel that, okay, well, that’s awesome. It’s not that bad … maybe I can manage this other way [p10]”. This timely access to appropriate conservative management advice was seen as preferable to a long wait for a formal clinic appointment: “I would prefer to get some advice, even if it’s from my GP … instead of waiting three years (for an appointment) [P3]”. Other perceived benefits included the time savings and reduced travel: “saves time, saves going to hospital with the traffic and paying for parking [P5]”.

Although there were several positives, some patients reflected their preference for seeing a specialist, primarily for reassurance: “how I see it as a patient, how I see it is, I need to see a specialist [P4]” and “I would’ve said for reassurance and for you to explain to be a lot better with your experience [P6]”.

The primary recommendation for improvement in the SVC from patients was for improved communication with the patient, particularly to inform the patient that advice had been provided to their referring doctor. Patients suggested that we “send one (letter) to the GP and then, send something to the client and say, we’ve sent a letter to the GP [P2]” or that “if you send something to the GP, send the same thing to the patient as well [P3]”. This recommendation appears to parallel the earlier insights offered by the patients regarding the limited feedback provided by their GP following receipt of the customised advice.

## 4. Discussion

This study explored the implementation and outcomes of a physiotherapy-led Spinal Virtual Clinic (SVC) model within a metropolitan public hospital setting. The findings from Stage 1 demonstrate that most referrals triaged to the SVC involved individuals presenting with low back pain, with or without leg symptoms, and that most were successfully managed through virtual correspondence without requiring a face-to-face appointment. Only a small proportion (5%) were identified as needing formal outpatient review, and even fewer ultimately proceeded to surgical intervention. The advice provided to referring GPs frequently included evidence-based conservative management strategies such as physiotherapy-led active care, neuropathic medication, and spinal injections. Notably, there were no cases of missed pathology requiring urgent surgical intervention, suggesting that the SVC process appeared clinically safe in this context. Furthermore, re-referral pathways were clearly outlined in the majority of cases, supporting continuity of care in the event of symptom progression. Semi-structured interviews with patients highlighted both the perceived value of the SVC, such as timely access, reassurance, and clear management plans, as well as areas for improvement, particularly regarding communication and patients’ understanding of the triage process. These findings highlight the potential of the SVC model to effectively triage and manage spinal referrals in a timely, resource-efficient manner while maintaining patient safety, and identifying areas of improvement through continuous quality improvement.

This research identified through a virtual clinic model that the majority of low acuity spinal patients can be managed safely in the community and do not require formal spinal surgical consultation. This finding adds to a growing body of work that promotes appropriate conservative management strategies in primary care and outlines clear guidelines for the role of imaging and indications for specialist review in this cohort of patients both in Australia [32] and internationally [33]. This virtual model promotes early identification of such referrals as well as engagement with referrers and is in stark contrast to the traditional outpatient specialist model approach that consigns these referrals to long-term waitlists. Audits reveal this remains an Australia wide problem [5] with delays well beyond clinically recommended timeframes for this patient cohort.

There are appreciable benefits of early access to input and advice in this cohort of patients that have traditionally been allocated to outpatient waitlists with long timeframes for specialist assessment. These benefits include early reassurance, early engagement with primary care and early identification of the small subgroup of patients in this cohort for whom further imaging, extra clinical information or early formal review is required. New models of care which provide alternative pathways for patients can improve access and reduce wait times for formal surgical appointments [34], with reduced wait times contributing to better health outcomes [3,35]. Virtual clinics offer an alternative solution for specialist clinics burdened by increased clinical demand and long wait times for appointments. Redirecting low acuity referrals back to primary care with a targeted management plan avoids patients being placed on long wait lists whilst improving the overall clinic capacity such that high acuity patients requiring specialist input can receive a timely appointment.

It is also important to note that some patients may need, or progress to, spinal surgical interventions. This study demonstrated that APP may have the potential to identify many of this cohort at desktop review (5%). Established communication pathways with general practice also assisted in identifying another small cohort of patients, either via updated imaging or where new relevant clinical information was provided by the referring doctor. In considering the safety of the SVC model, it is important to reflect on the two cases of acute deterioration that occurred following their initial assessment. In both instances, the clinical decision made at that time of the SVC were based on presenting symptoms, examination findings, and available investigations, and consistent with established practice. In the absence of the SVC model, these patients, would have likely been placed on a standard outpatient waitlist, with no early clinical engagement or opportunity for timely escalation prior to their acute presentation. The SVC process incorporates several safety measures, including direct communication with the referrer, provision of advice regarding symptom monitoring, and clear pathways for urgent re-referral or emergency presentation. These features align with elements of other international referral review and discharge models, which emphasise early triage, targeted communication, and defined escalation protocols. Nevertheless, our findings highlight that acute deterioration may still occur despite these safeguards, reinforcing the importance of ongoing monitoring, clear escalation processes, and regular review of safety outcomes. Further investigation is warranted to assess long-term safety, patient-reported outcomes, and the comparative effectiveness of SVC against other models of care.

This study identified that patients value communication, and although the SVC produces comprehensive customised written clinical advice which is provided to the treating doctor in primary care, the initial iteration of this model of care did not produce formal communication to the patient. Clinician-patient communication can influence outcomes [36], and alerting the patient in writing to the provision of clinical advice to the GP may improve both access to the care recommended, as well as empower the patient to explore the clinical recommendations provided with their referring doctor. GPs have long been the gatekeepers in primary care [37], but in the rise of patient-centred care, this addition to the SVC model may facilitate patient-led review and implementation of the clinical advice which may enhance patients’ experiences of the SVC.

### Limitations

As with any research, there were limitations to this study. The first is that the data that formed the basis for this study reflect a small, although sequential cohort. Given the small sample size and single location, careful consideration is required regarding the generalisability of the results and transferability of the findings. In particular, the applicability of the SVC model to other clinical specialties or healthcare settings may be limited, as the effectiveness and feasibility of implementation could vary depending on local resources, referral processes, and clinician roles. Similarly, the sample may be subject to self-selection bias, with participants potentially more motivated to share either positive or negative experiences, and may not represent the perspectives of patients who chose not to participate. Given that the study did not purposively include individuals who were re-referred or underwent surgery following SVC discharge, this may further limit the representativeness of the findings. Telephone interviews, while necessary due to COVID-19 restrictions, may have limited rapport or depth compared with face-to-face interviews. Future research could evaluate a larger cohort of patients, and a comparative trial could evaluate outcomes of the SVC against more traditional models of care, including a model where the patients are seen by the APP. Secondly, longer-term follow-up of this cohort of patients may have identified more cases that proceeded to an index spinal procedure. The cut-off date of 31 December 2021 enabled the research team to have a finite date so that the original data could be presented in a timely manner. A minimum timeframe of six months post referral we believed would likely capture any referrals with acute neuro-compressive lesions that might have been missed. Longer-term follow-up of this cohort, with larger sample sizes, could be considered in the future research, although subsequent surgical rates may be confounded by new pathology that may not have meant original triage was inaccurate or inappropriate. Thirdly, it is possible that some patients may have sought a surgical opinion in the private sector. Our audit of further public hospital involvement up to 31st December 2021 would not have captured any subsequent surgery undertaken in the private sector. As apparent in the nine surgical cases, one was undertaken in the private sector and only identified due to admission to our facility with a complication. Although this study provides some emerging evidence on this model of care, future research could examine GP perspectives. Evaluation of the SVC model across multiple sites, specialties, and patient populations would be essential to better understand its generalisability and to determine whether its benefits can be replicated in diverse clinical contexts. For example, future studies could consider stratified sampling to explore differences in patient experience based on subsequent care trajectories following SVC discharge. Evaluation of an SVC in other jurisdictions may assist in examining the transferability of this model of care and consideration of a prospective trial to evaluate long-term outcome for patients as well as investigating the relative cost benefits of this model.

## 5. Conclusions

This research provides emerging evidence on the impact and patient perspectives of a model of care implemented for the first time in South Australia—the Spinal Virtual Clinic. Most referrals triaged as low acuity can be managed in primary care, and physiotherapists working in advanced practice are well-placed to identify patients who may benefit from formal surgical review. The findings highlight the potential for the SVC model to streamline outpatient triage by identifying patients who may be suitable for management in primary care with tailored advice. In the context of increasing demands on outpatient services, this model could contribute to service redesign in other high-demand specialties facing lengthy waitlists and limited clinic capacity. While these findings suggest the model can facilitate timely and appropriate care, the small number of acute deteriorations observed underscores the importance of robust safety measures, clear escalation pathways, and ongoing evaluation of outcomes.

## Figures and Tables

**Table 1:** Referral characteristics.

Diagnostic Group	n	Percentage of Total
Low back pain with leg pain	136	42.9%
Low back pain	65	20.5%
Neck pain with arm pain	35	11.0%
Thoracic pain/scoliosis	19	6.0%
Neck pain	16	5.0%
Spinal pain (multiple areas)	11	3.5%
Insufficiency fracture	5	1.6%
Claudicant leg symptoms	3	0.9%
Other (details)	27	8.5%

**Table 2:** Summary of SVC advice provided.

Advice Recommended	n	Percentage of Total
Physiotherapy/active management	256	80.8%
Neuropathic medication	113	35.6%
Spinal injection	111	35.0%
Further investigation	42	13.2%
Simple analgesia	26	8.2%
Alternative medical specialist	24	7.6%
Appointment booked	3	0.9%
Other	39	12.3%

**Table 3:** Interviewed patient characteristics.

ID	Age	Gender	Employment Status	Medical Complaint(s)
P1	66	M	Aged pension	Low back pain; aching/restless legs; groin pain
P2	42	F	Unemployed	Low back pain/spasm; leg numbness
P3	27	F	Employed	Neck and shoulder pain; low back pain/numbness
P4	56	M	Unemployed	Back pain; problems with walking & standing/leg numbness; previous back surgery
P5	65	F	Employed	Spinal issues; leg numbness/tingling; arthritis in ankles and neck
P6	64	F	Employed	Neck pain; osteoarthritis
P7	33	F	Employed	Back pain
P8	76	F	Retired	Back pain; compression fractures; osteoporosis
P9	47	M	Employed	Back pain; anterior cruciate ligament reconstruction
P10	61	F	Disability pension	Back pain; blocked arteries/pain in the legs
P11	75	F	Retired	Back and leg pain; osteoarthritis

## Data Availability

All relevant data have been reported in this manuscript. Additional data can be sourced by contacting the authors.
